# Treatment of ocular tumors through a novel applicator on a conventional proton pencil beam scanning beamline

**DOI:** 10.1038/s41598-022-08440-5

**Published:** 2022-03-17

**Authors:** Rajesh Regmi, Dominic Maes, Alexander Nevitt, Allison Toltz, Erick Leuro, Jonathan Chen, Lia Halasz, Ramesh Rengan, Charles Bloch, Jatinder Saini

**Affiliations:** 1 Department of Radiation Oncology, University of Washington School of Medicine, 1959 NE Pacific St., Seattle, WA 98195 USA; 2 Radiotherapy Physics Department, UCLH NHS Foundation Trust, 5th Floor West, 250 Euston Road, London, NW1 2PG UK

**Keywords:** Cancer, Health care, Physics

## Abstract

Treatment of ocular tumors on dedicated scattering-based proton therapy systems is standard afforded due to sharp lateral and distal penumbras. However, most newer proton therapy centers provide pencil beam scanning treatments. In this paper, we present a pencil beam scanning (PBS)-based ocular treatment solution. The design, commissioning, and validation of an applicator mount for a conventional PBS snout to allow for ocular treatments are given. In contrast to scattering techniques, PBS-based ocular therapy allows for inverse planning, providing planners with additional flexibility to shape the radiation field, potentially sparing healthy tissues. PBS enables the use of commercial Monte Carlo algorithms resulting in accurate dose calculations in the presence of heterogeneities and fiducials. The validation consisted of small field dosimetry measurements of point doses, depth doses, and lateral profiles relevant to ocular therapy. A comparison of beam properties achieved through the applicator against published literature is presented. We successfully showed the feasibility of PBS-based ocular treatments.

## Introduction

Proton therapy is widely used for the treatment of ocular (including intra-ocular) tumors^1–3^ with excellent control rates^4–6^. Although access to proton therapy has increased greatly with the opening of many of clinical proton therapy centers globally, this has not translated to increased access to proton-based ocular treatments. This is because most ocular treatments are performed on dedicated proton beamlines with specific beam properties and most newly constructed proton therapy facilities are not equipped with an ocular-specific beamline. As most ocular targets are small and shallow requiring the proton beam range from 0 to 5 cm, dedicated proton beamlines are generally based on accelerator systems that can produce low energy proton beams (i.e. ranges < 5 cm) without requiring extra energy degradation. This results in sharp distal and lateral dose fall-offs, lending these systems dosimetric superiority. Furthermore, the snout footprints of dedicated ocular beamlines are generally small enough to allow for placement of patient-specific apertures close to the surface maximizing the lateral penumbra advantage. Conventional proton beamlines are designed for the treatment of larger fields and have multiple limitations: First, due to reduced dose rate at lower energies, these systems have a lower limit on the proton energy at the nozzle exit. Treatment of ocular targets would require an energy degrading device (range shifter) in the beam path that has the impact of broadening distal and lateral penumbras. Secondly, the footprint of the beamlines at the exit plane requires a large air gap (> 12 cm) between the patient surface and snout exit further broadening the lateral penumbra. The goal of this work is to address this second limitation and present the design, commissioning, and validation of an ocular applicator that mounts on a conventional proton snout. The proposed applicator reduces the footprint of the beamline allowing for treatment with short air gaps (~ 4 cm) like dedicated beamlines maximizing the lateral penumbra advantage. The achieved values of lateral and distal penumbra are comparable to published literature as discussed in the subsequent sections.

There are implications of our work for newly opened proton therapy centers to offer proton treatments for ocular targets without investing in a separate beamline. Furthermore, our solution utilizes modern advanced treatment planning and delivery technologies to be used for ocular proton therapy. Pencil beam scanning (PBS) has been emerging as a modality of choice for proton therapy as it affords the clinicians the ability to shape dose in 3 dimensions. The proposed applicator works with PBS providing potential normal tissue sparing through inverse planning techniques. Another advantage is the ability to employ Monte Carlo algorithms for treatment planning providing accurate dose estimation. Traditional dedicated ocular beamlines are based on obsolete delivery techniques such as double scattering and employ analytical algorithms, which are shown to be inaccurate^7,8^ when proton beam traverses through implanted markers and inhomogeneous media.

Previous work by Ciocca^9^ showed the feasibility of performing ocular treatments through pencil beam scanning on a synchrotron-based facility. In their study, MC simulations in Fluka^10^ were performed to calculate optimum range shifter and collimator positions. The best beam properties (sharpest lateral dose fall-off) were found when the range shifter was placed upstream of the beam monitoring ion chambers. However, placing range shifters upstream of beam monitoring ion chambers is not feasible for most clinical facilities as that would mean substantial changes to the beamline, which may not be allowed by the proton vendor. Additionally, their work was limited to non-commercial FLUKA MC rather than FDA-approved commercial clinical MC. In another study by Slopsema et al.^11^, the development of a proton beamline is demonstrated for a cyclotron-based proton facility. However, this work was done for a scattering system rather than a PBS system which is the more common modality among newly constructed proton facilities. In this work we present the design and clinical validation of a novel applicator compatible with an IBA proton pencil beam scanning beamline^12^ to allow treatment of ocular targets. Utilizing the RayStation planning system, we showed that no separate commissioning measurements are required as an acceptable agreement is achieved with a planning system commissioned for large clinical fields. The goals of this paper are as follows:

- Presentation of a novel proton PBS applicator with design parameters optimized for treatment of ocular targets.
- Commissioning and clinical validation of the applicator for treatment.
- Comparison of relevant beam properties between the presented system and available results of dedicated ocular beamlines.

## Materials and methods

### Equipment and instrumentation

The Seattle Cancer Care Alliance Proton Therapy Center (SCCAPTC) is a four-room proton therapy facility^13^ with capabilities to treat patients using PBS and uniform scanning (US). The center is equipped with the IBA Proteus Plus (Ion Beam Applications, Louvain-La-Neuve, Belgium) system. Patients are simulated with a GE Optima 580 W CT scanner (GE Healthcare, Waukesha, WI). MOSAIQ (version 2.64) (Elekta Medical System, Sweden) oncology information system is used to deliver and track patient treatments. No patients were involved in this study. For this work, treatment planning and validation was performed with RayStation version 9B (RS) TPS^14^. Depth dose measurements were performed with a commercial microdiamond detector (Type 60019, PTW-Freiburg, Germany) and PC electrometer (Sun Nuclear Corp., Melbourne, FL). Two-dimensional dose profile measurements were made through Gafchromic EBT3 film (Ashland Advanced Materials, NJ) and analyzed through DoseLab software (Varian Medical System, Inc., CA). For end-to-end testing, the phantom localization and set up were performed with planar orthogonal X-ray images that were acquired and analyzed through AdaptInsight Imaging System (Ion Beam Applications, Belgium). Patient-specific 2-D dosimetric measurements were acquired through EBT3 film with analysis performed in OmniPro I’mRT (Ion Beam Applications, Louvain-La-Neuve, Belgium) software.

### Design of ocular applicator

**Figure 1:**
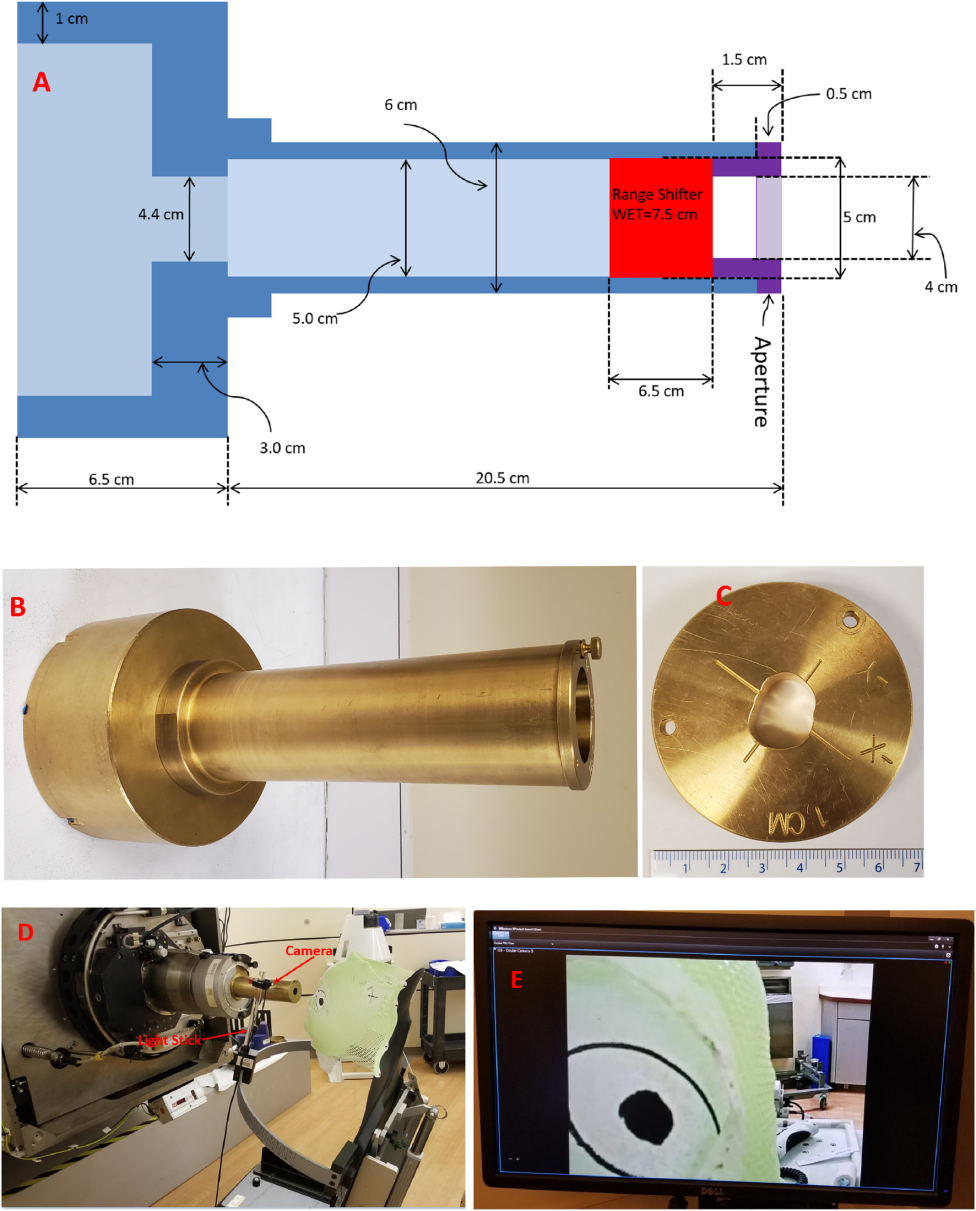
(**A**) Schematic of the applicator with relevant dimensions (not to scale). (**B**) An actual picture of the applicator. (**C**) A custom patient-specific aperture. (**D**) Light stick with camera mount to monitor the gaze. (**E**) Field of view of camera while monitoring gaze. The aperture is held to the applicator through a thumbscrew.

Figures 1A and B show the schematic and actual picture of the applicator. The applicator is made from brass and is mounted to the smallest snout (10 cm snout) with the same mounting clips used to mount apertures for conventional treatments. The applicator has an built-in acrylic range shifter that corresponds to a physical thickness of 6.5 cm and water-equivalent-thickness of 7.5 cm. The range shifter is needed as the lowest proton beam energy available at the nozzle exit is 98.5 MeV that corresponds to proton beam range of 7.5 cm in water. A custom patient-specific aperture (Fig. 1C) can be mounted at the end of the applicator to enhance lateral dose conformality. The applicator can accommodate a maximum circular opening of 4 cm. The capabilities of the applicator are given in Table 1.

**Table 1:** Specifications of the ocular applicator.

Applicator property	Value
Maximum proton beam range @ Nozzle plane (cm)	15
Minimum proton beam range @ Nozzle plane (cm)	7.5
Fixed range shifter WET (cm)	7.5
Fixed range shifter physical thickness (cm)	6.5
Range shifter material	PMMA
Maximum proton beam range after range shifter (cm)	7.5
Minimum proton beam range after range shifter (cm)	0
Maximum field size circular diameter (cm)	4
Patient specific aperture material	Brass
Patient specific aperture physical thickness (cm)	1.5

The given applicator was commissioned into a commercial treatment planning system RayStation version 9BSP1. RayStation physics module allows the user to define relevant geometry of the applicator in terms of inner and outer dimensions, and order of beam line elements such as range shifter and aperture.

A couple of factors guided the design of the applicator. The planner can minimize the lateral penumbra by placing the beam collimating aperture close to the patient's surface. With conventional snout provided by the vendor, the body of the snout has a larger footprint preventing short air gaps. We run into collisions between snout housing and patient body at our clinic, necessitating large air gaps (~ 8–10 cm). For ocular treatment, we utilize a camera-mounted light stick that moves on a semi-circular aluminum bar plate (see Fig. 1D) for eye-gaze fixation and monitoring. The light stick slides on the aluminum bar plate depending on the desired patient gaze angle (i.e., neutral, temporal, or nasal). Additionally, we can move the camera position vertically to move the gaze up or down as needed. The patient is asked to focus on a dot that is painted at the tip of the camera. The light stick and aluminum bar plate have indexing engraved to allow for reproducible gaze fixation during the simulation and treatment. Our radiation therapists are able to monitor the gaze in real-time during the treatment through the camera mounted on the light stick. Figure 1E shows field of view of camera displayed on the computer screen during the treatment. This gaze-fixation technique requires patients to have an open field of view to the camera. Conventional snouts can obscure patients' fields of view due to their large footprints, requiring planners to increase the air gap further. Our proposed applicator has a cylinder tube of only 6 cm, allowing us to place the aperture very close to the patient surface (air gaps of 3–5 cm), ensuring that eye-gaze fixation is successful and maintaining the penumbra.

### Validation measurements

#### Depth dose comparison

Depth dose measurements for Bragg peaks (single layer monoenergetic scanned fields) were carried out for three different circular apertures of 1.5 (F1.5), 2 (F2), and 3 (F3) cm diameter. These fields are composed of spots of single proton energy arranged in a grid-like pattern with an inter-spot spacing of 0.2 mm in x- and y-directions to achieve a laterally uniform dose distribution. The spot patterns for these fields were created to over-scan the respective aperture size. For each aperture size, three beams were created corresponding to a proton beam range of 2 cm, 4 cm, and 6 cm in water for a total of 9 beams. The measured depth doses were generated from individual point-by-point dose measurements along the central axis with a microdiamond detector.

For comparisons, the measurement geometry for beam and set-up were simulated in the RS TPS through forward planning technique, and calculated depth doses at the central axis were exported. All RS calculations were performed with an MC dose calculation algorithm with a statistical uncertainty of less than 0.5% and an isotropic grid size of 1 mm was used for calculations in the TPS.

Measurements were also made for spread-out Bragg peaks (SOBP) in a water phantom. These beams were generated in the RS TPS through inverse planning by optimizing dose to cylindrical targets and utilizing apertures for sharper lateral dose fall-off. The cylindrical targets of diameters 1.5, 2 and 3 cm were created to have a circular footprint in beams eye view (BEV) corresponding to the aperture sizes of 1.5, 2 and 3 cm respectively. The height and position of the target inside the water phantom were chosen to have two range-modulation combinations: (1) range = 2 cm, modulation = 1.5 cm, and (2) range = 6 cm, modulation = 3 cm. The combination of aperture size, height, and the targets resulted in a total of 6 beams. Once optimized to deliver a uniform dose to the targets, the beams were transferred to Mosaiq and delivered into a water phantom. Like single layer fields, the measured depth doses were generated from individual point-by-point dose measurements along the central axis of each beam with a microdiamond detector.

The analysis for single layer and SOBP beams consisted of point-by-point dose difference between measurements and calculated depth doses. The magnitude of point-by-point dose differences was averaged for all the points along the depth doses and results were considered acceptable if the difference was less than 2%. For SOBP beams, a tolerance of ± 3% was employed for point dose difference at any point along the depth dose except at the distal edge. Additionally, range error between calculated and measured depth doses was evaluated by comparing R80 depths (distal depth corresponding to 80% of maximum) with a tolerance of 1 mm.

#### Profile comparison

2-D profile measurements were also obtained using EBT3 film sandwiched inside proton-compatible solid water for every single layer and SOBP beam from the previous section. For single-layer beams, the measurements were performed at two depths: (1) at the entrance region and (2) near the Bragg peak. For SOBP beams, the profile measurements were made at the center of SOBP. The irradiated EBT3 films were scanned 24 h post-exposure on a flatbed scanner (Epson Expression 11000XL, Epson America Inc., California, USA) using 72 pixels per inch of resolution and landscape orientation. A control film that was similarly handled but not exposed was also scanned to obtain net optical density using the red channel. The films were compared to calculated dose profiles from the treatment planning system through gamma index (GI) analysis. A dose threshold of 2%, distance tolerance of 1 mm, and low dose threshold of 5% were used. In total, 24 beam profiles were measured and analyzed.

#### Point dose comparison

A comparison of absolute dose output between measured and calculated fields for the single-layer and SOBP fields was performed. For SOBP beams, the point dose measurements were made at the center of the SOBP for all three field sizes. The combination of three field sizes along with two range-modulation sets resulted in a total of 6 measurements. For single-layer uniform fields, the point dose measurements were made at the entrance region at a depth of 5 mm. Like SOBP beams, all three circular field sizes were employed for three ranges i.e. 2 cm, 4 cm, and 6 cm resulting in a total of nine beams. The point dose measurements were performed using a diamond detector cross calibrated against an ADCL calibrated parallel plate chamber. The cross calibration was done at field size of 10 × 10 cm^2^ using range 16 cm and modulation 10 cm. A tolerance of 2% was used for this analysis.

#### Applicator alignment

Although PBS allows for lateral collimation of the beam through the placement of spots conforming to the target extents, it has been shown that a sharper lateral dose fall-off^15,16^ could be achieved by a patient specific aperture. The use of aperture for PBS adds another layer of complexity for isocenter verification. For double scattering and uniform scanning-based proton therapy, the proton beams over-scan the aperture requiring only the mechanical isocenter of the patient-specific beam modifying devices (aperture and compensators) to coincide with the imaging isocenter. For aperture-based PBS treatments, the congruency of all three isocenters i.e. imaging, mechanical, and proton beam needs to be validated. We performed a film exposure test to measure the discrepancy between all three isocenters and evaluate any changes due to applicator travel along the beam axis. A EBT3 film with fiducials was aligned to the imaging isocenter through the in-room orthogonal X-ray system. Once the film was aligned using X-rays, we put the aperture into the snout and irradiated the film. Exposure of the film was obtained with a 4 cm aperture in the beam path. The PBS beam consisted of two layers to obtain an aperture outline and central spot alignment on the EBT3 film. The first layers consisted of a low dose uniform lateral profile that over-scanned the 4 cm aperture. The second layer consisted of a single spot of high intensity centered on the central axis. Once exposed, the film allowed the extraction of relative error in all three isocenters: (1) imaging iso at the intersection of fiducials, (2) mechanical iso at the geometric center of low dose exposure from the first layer and (3) proton beam iso at the centroid of the single spot exposure from the second layer. The test was conducted for five snout positions corresponding to air gaps: 1 cm, 5 cm, 11 cm, 15 cm, and 20 cm by retracting the snout. The analysis consisted of evaluating relative x- and y-spatial errors between the different isocenters.

#### End to end test

An end-to-end test was conducted using the clinical protocols of CT simulation, imaging, and treatment delivery. Treatment for ocular targets is conducted in a chair at SCCAPTC as described here^17^. A phantom was constructed out of blue wax^18^ in a cylindrical shape with an insert to place film at 2 cm depth. The phantom was scanned on a GE Optima CT 5580 using the ocular protocol corresponding to a field-of-view of 50 cm and slice thickness of 1.25 mm. A pseudo target (Fig. 7A) that roughly represents a clinical ocular target at our clinic of roughly 0.5 cm width, 0.8 cm length, and 0.4 cm thick was created at the depth of 2 cm. A single anterior oblique beam with a custom aperture was inversely optimized to deliver a 50 Gy RBE dose to the target in the RS planning system. The optimized plan was transferred to Mosaiq OIS for treatment delivery. The phantom with a EBT3 film was aligned at the isocenter through the AdaptInsight imaging system. The film was compared to the corresponding dose plane from the planning system using the GI analysis. A tolerance of 1% dose difference, 1 mm distance-to-agreement, and 10% low dose threshold were used. The absolute dose at the central axis of the field was also measured through a calibrated pin-point ion chamber and compared to corresponding value from the TPS with a tolerance of 3%.

#### Simulated lateral and distal penumbras

The dose fall-off properties of the proton beam in lateral and distal directions are important for ocular targets that are generally small and have numerous critical OARs in the vicinity. Most ocular specific beamlines have lower proton beam energies (~ 55–100 MeV) requiring minimal compensation to produce a clinically relevant beam of range 1–4 cm. By minimizing material in the beam path, these systems can produce relatively sharp lateral and distal dose fall-offs^11^. The lowest PBS beam energy at SCCAPTC is 98.5 MeV which corresponds to the proton beam range of 7.5 cm in water. For treatment of ocular targets, beam energy must be degraded to a clinically relevant range and thus our proposed ocular snout has a fixed range shifter of 7.5 cm WET. Introducing this range shifter in the beam path degrades both distal and lateral penumbra. To compare distal and lateral penumbras presented in this study to available literature^8,11,19^, we simulated relevant ocular beams in the TPS with various range-modulation combinations. Seven beams were created to give a uniform dose to a box target of 1.5 cm in diameter. A beam shaping aperture was used to sharpen the lateral penumbra. Distal and lateral penumbra values were calculated from 80 to 20% dose fall-off from the relevant dose planes. A brief comparison between our penumbra values against values from published literature is presented in the discussions section.

## Results

### Validation measurements

#### Depth doses

**Figure 2:**
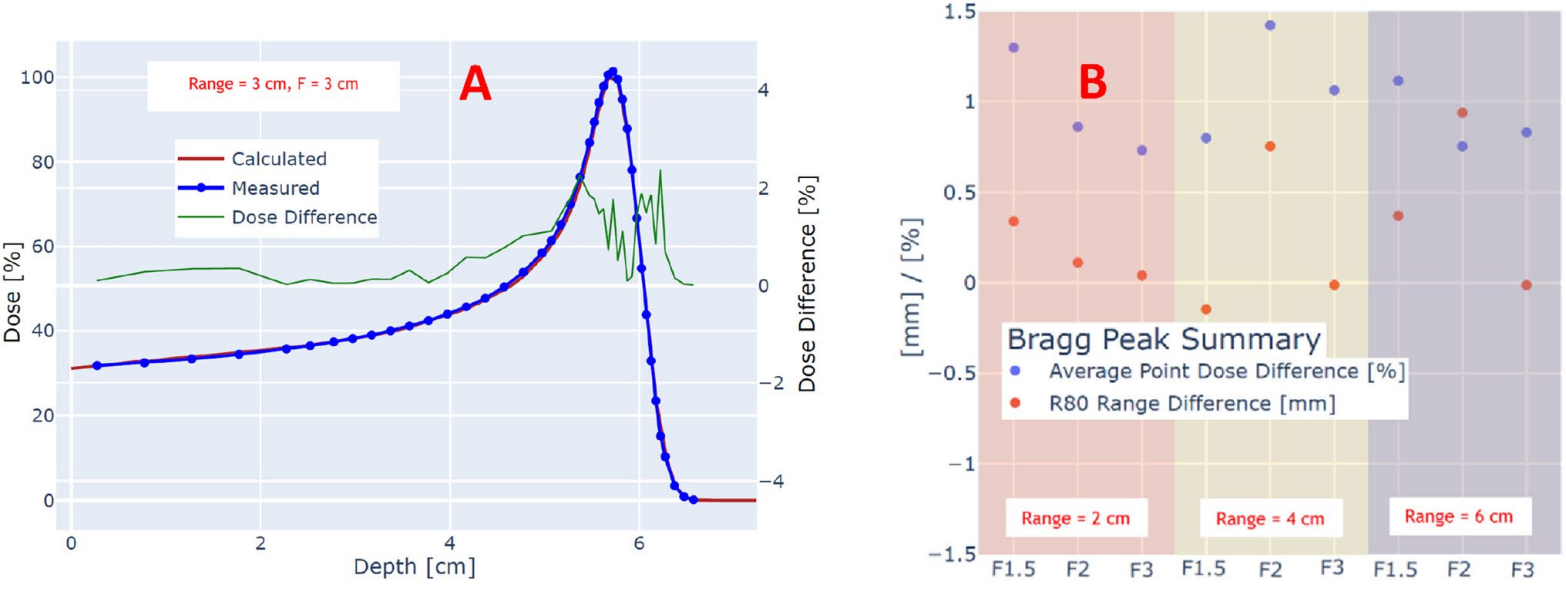
(**A**) Depth dose comparison between measured and calculated Bragg peak with a range of 3 cm and circular field size of 3 cm. The local error between measurement and calculated is shown with a green line. (**B**) Average point dose and R80 range differences for ranges 2, 4, and 6 cm as a function of circular field size.

Figure 2A shows an example of depth dose comparison between measured and calculated for a Bragg peak with range of 6 cm, and circular field size of 3 cm. The maximum deviation for point-by-point dose difference is 2.2%. The absolute magnitudes of point-by-point dose differences were averaged along the depth dose and shown for all Bragg peaks in Fig. 2B. The maximum absolute averaged point dose difference is 1.4% for beam with a range of 4 cm, and circular field size of 2 cm. The maximum range discrepancy between measured and calculated Bragg peaks as evaluated through R80 depth is 0.9 mm for Bragg peak with range of 6 cm, and circular field size of 2 cm, with all the range error between the interval of − 0.2 to 0.9 mm.

**Figure 3:**
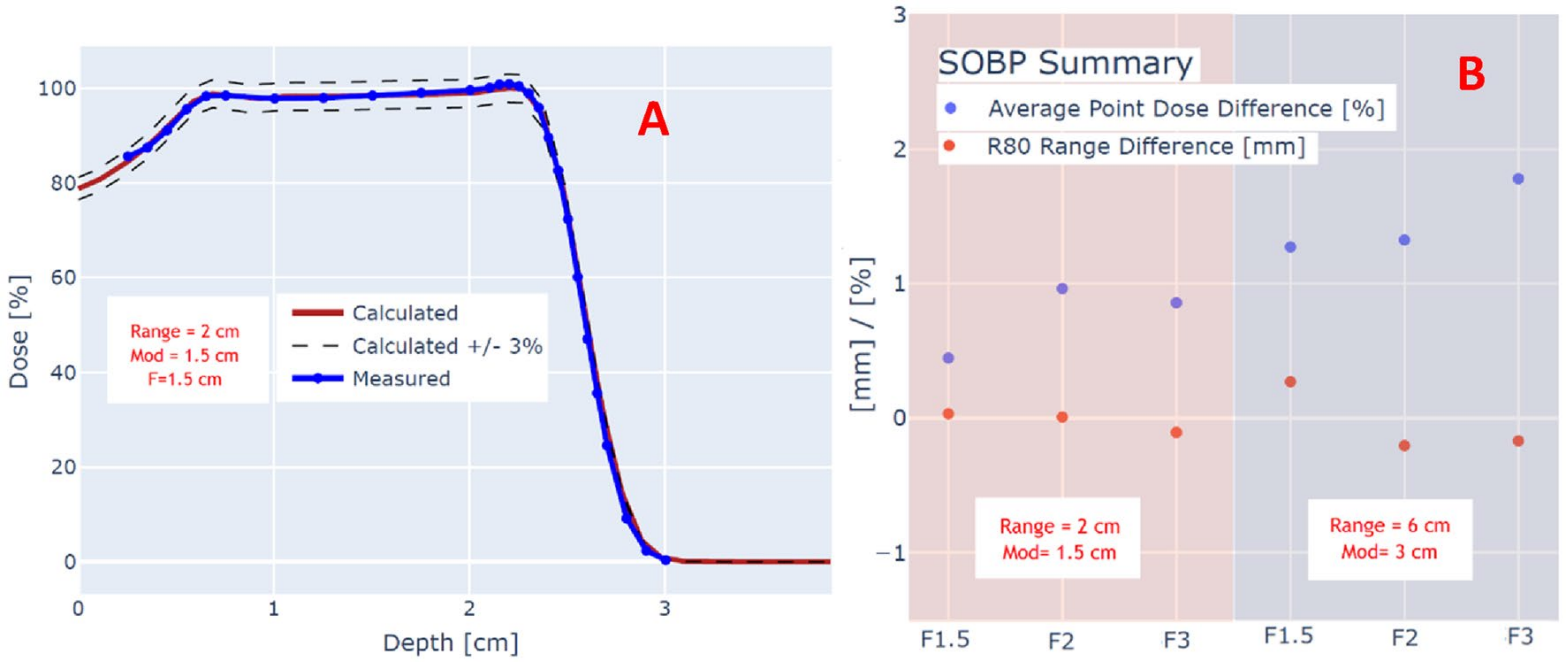
(**A**) Depth dose comparison between measured and calculated SOBP with a range of 2 cm, modulation of 1.5 cm, and circular field size of 1.5 cm. The ± 3% error bars are shown as dotted lines along with the depth dose. (**B**) Average point dose and R80 range differences for SOBP beams as a function of circular field size.

Figure 3A shows a comparison of a SOBP beam with a range of 2 cm, modulation of 1.5 cm, and circular field size of 2 cm. The dotted lines represent the ± 3% local dose difference between measured and calculated depth doses. For all the six SOBP beams, the maximum point-by-point dose difference at any point along the depth dose was less than 3%. Like Bragg peaks, the point-by-point dose differences were averaged for all points along the depth dose and plotted as a function of the beam name (Fig. 3B). The absolute averaged point dose errors were within 0.4–1.8%. Figure 3B also shows the SOBP beam range errors with a maximum discrepancy between measured and calculated to be 0.3 mm for a SOBP beam with range of 6 cm, modulation of 3 cm, and circular field size of 1.5 cm.

#### Profile comparison

**Figure 4:**
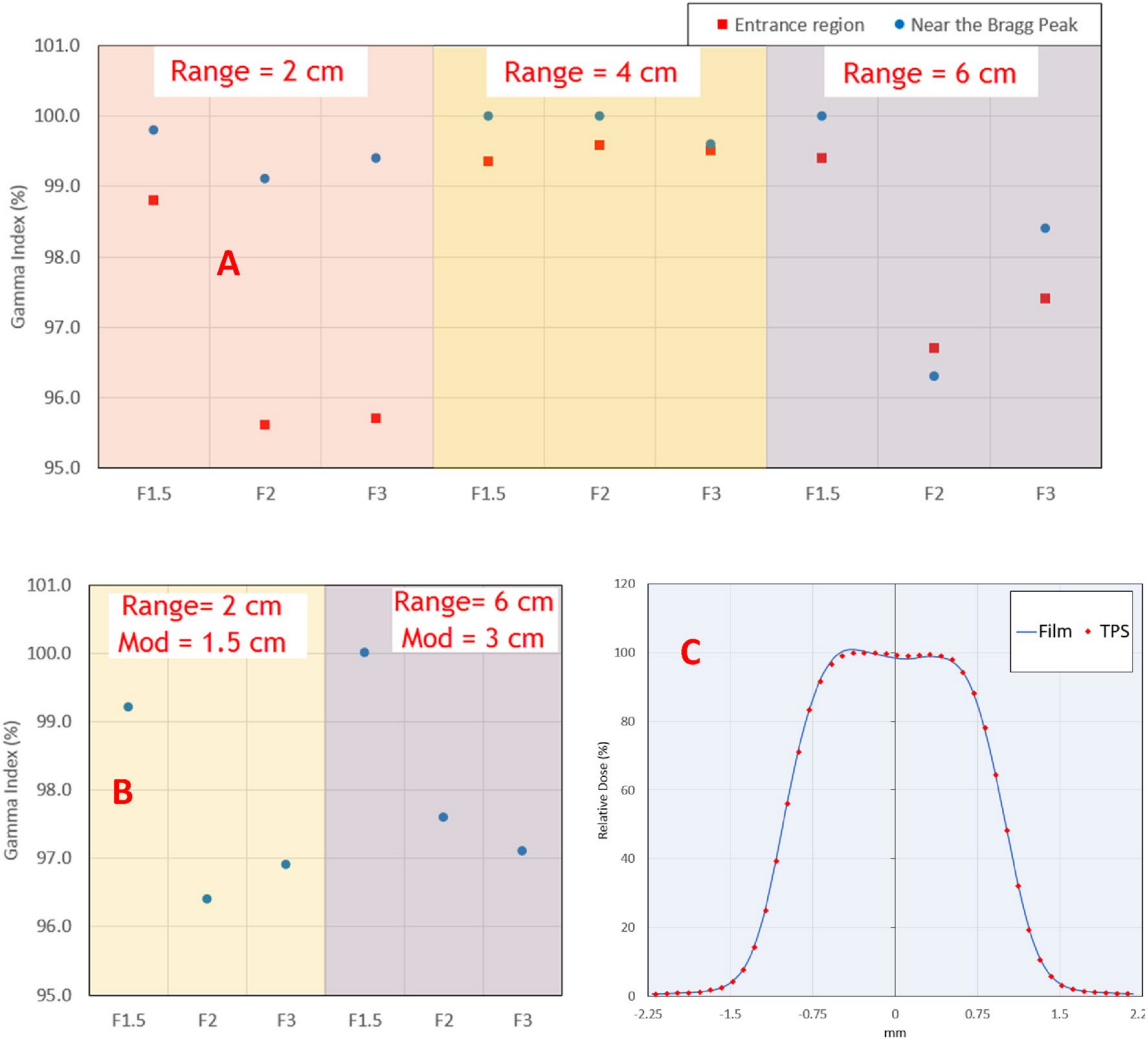
(**A**) Gamma Index analysis for Bragg peak beams at the entrance region and a depth near the Bragg peak, as a function of the field size. (**B**) Gamma Index analysis for SOBP beams at depths corresponding to the center of the SOBP as a function of the field size. (**C**) An example dose profile in the center of the SOBP for a field with a 2 cm circular aperture.

Figure 4 shows the results of GI analysis for Bragg peaks (A) and SOBPs (B). All the 24 dose planes had GI > 95%, with average GI of 98.6% and 97.9% for Bragg peaks and SOBPs respectively. An example of a dose profile comparison between film and planning system is shown in Fig. 4C for a field with a 2 cm circular aperture.

#### Point dose comparison

**Figure 5:**
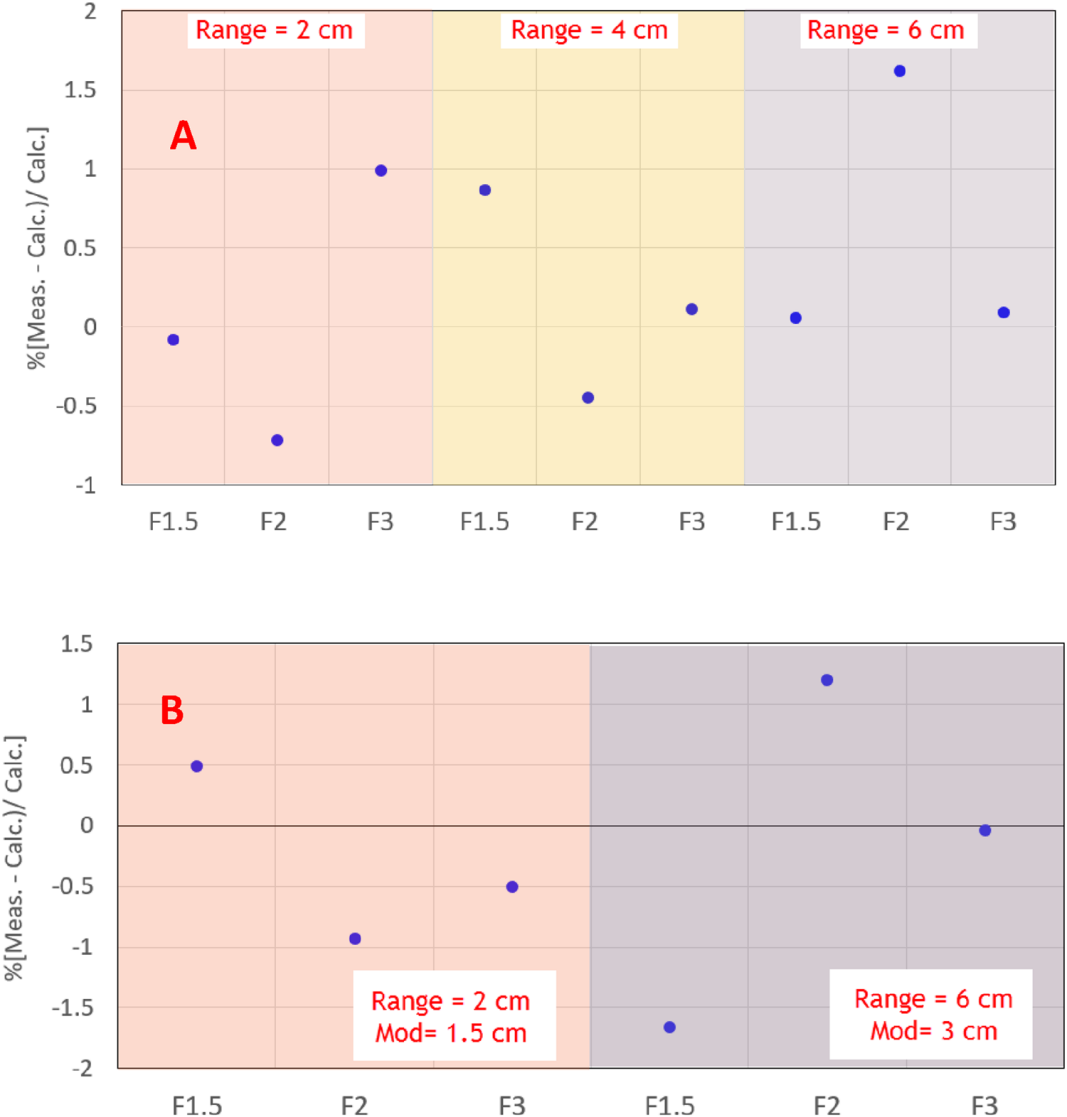
(**A**) Absolute point dose difference between measured and calculated depth dose around entrance region of three Bragg peaks as a function of circular field size. (**B**) Absolute point dose difference between measured and calculated depth dose around the center for SOBPs beams as a function of circular field size.

The absolute point dose comparison between measured and calculated for Bragg peaks with different field sizes is shown in Fig. 5A. There was no obvious pattern in variation of the output factor with the field size for any of the three ranges considered. All the calculated dose values were with ± 2% of the measured.

Figure 5B shows the point dose comparisons for measurements around the center of modulation for SOBP beams with varying field sizes. Like Bragg peaks, no discrepancy between calculated and measured point doses was observed with variation in the field size. The discrepancy between measured and calculated absolute point dose is within ± 2%.

### Applicator alignment

**Figure 6:**
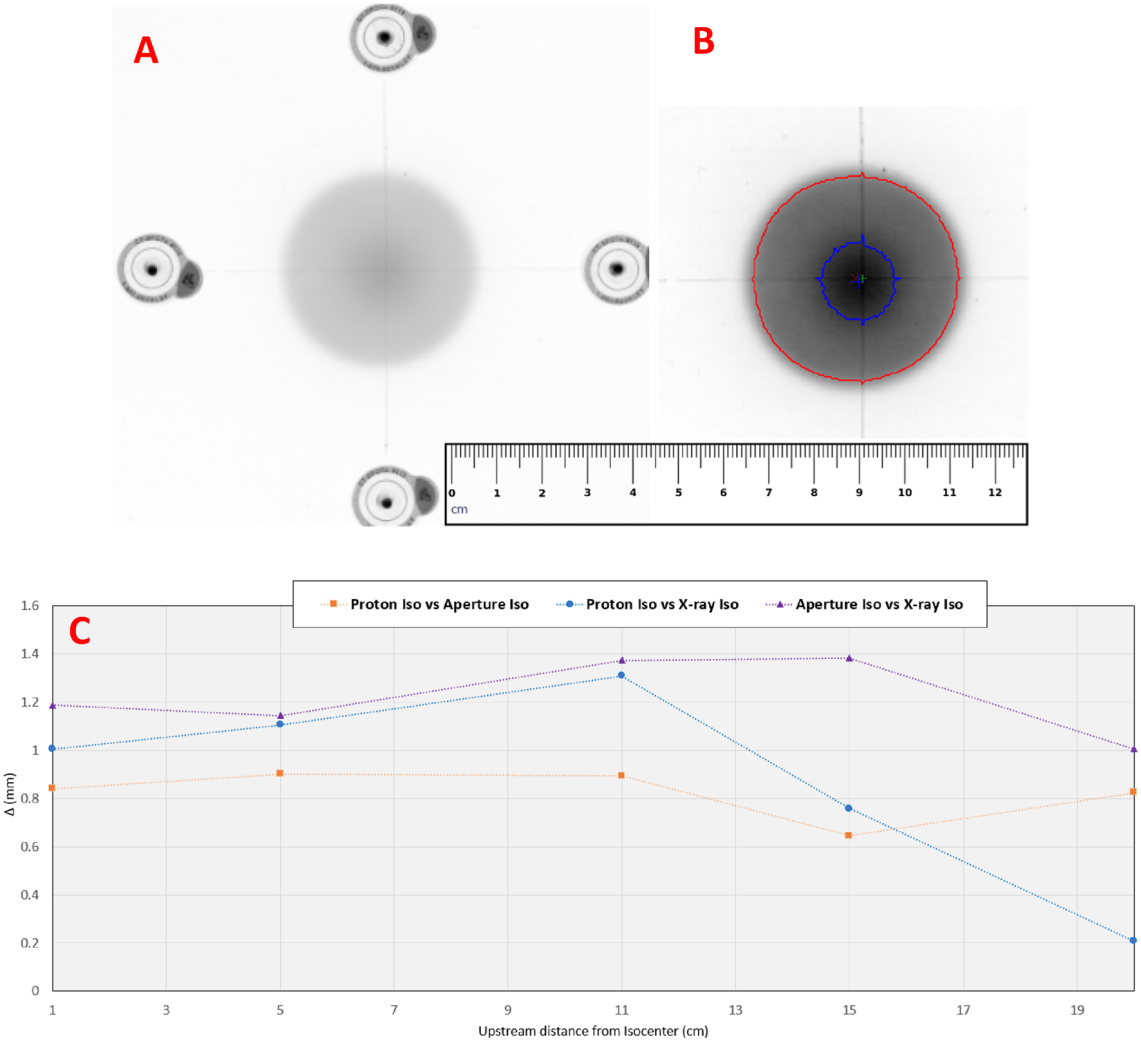
(**A**) An example of an irradiated film used to determine relative errors between imaging, proton beam, and mechanical isocenters. (**B**) Resulting film analysis with proton beam isocenter (blue cross), mechanical isocenter (red cross), and imaging isocenter (green cross). (**C**) Relative alignment errors between proton beam, X-ray, and aperture isocenters as a function of upstream distance from the isocenter plane. The measurement points are shown by markers, the connecting lines are for visualization purposes only.

Figure 6 shows the relative alignment errors between proton beam, X-ray, and aperture isocenters as a function of upstream distance from the isocenter plane. The maximum errors between (1) proton beam and aperture central axis, (2) proton beam and X-ray iso, and (3) aperture central axis and X-ray iso were 0.9 mm, 1.3 mm, and 1.38 mm, respectively. As most treatments for ocular targets would occur at small air gaps corresponding to aperture positions of 1–5 cm, the effective error between all three isocenters in this range was below 1.2 mm.

### End-to-end test

**Figure 7:**
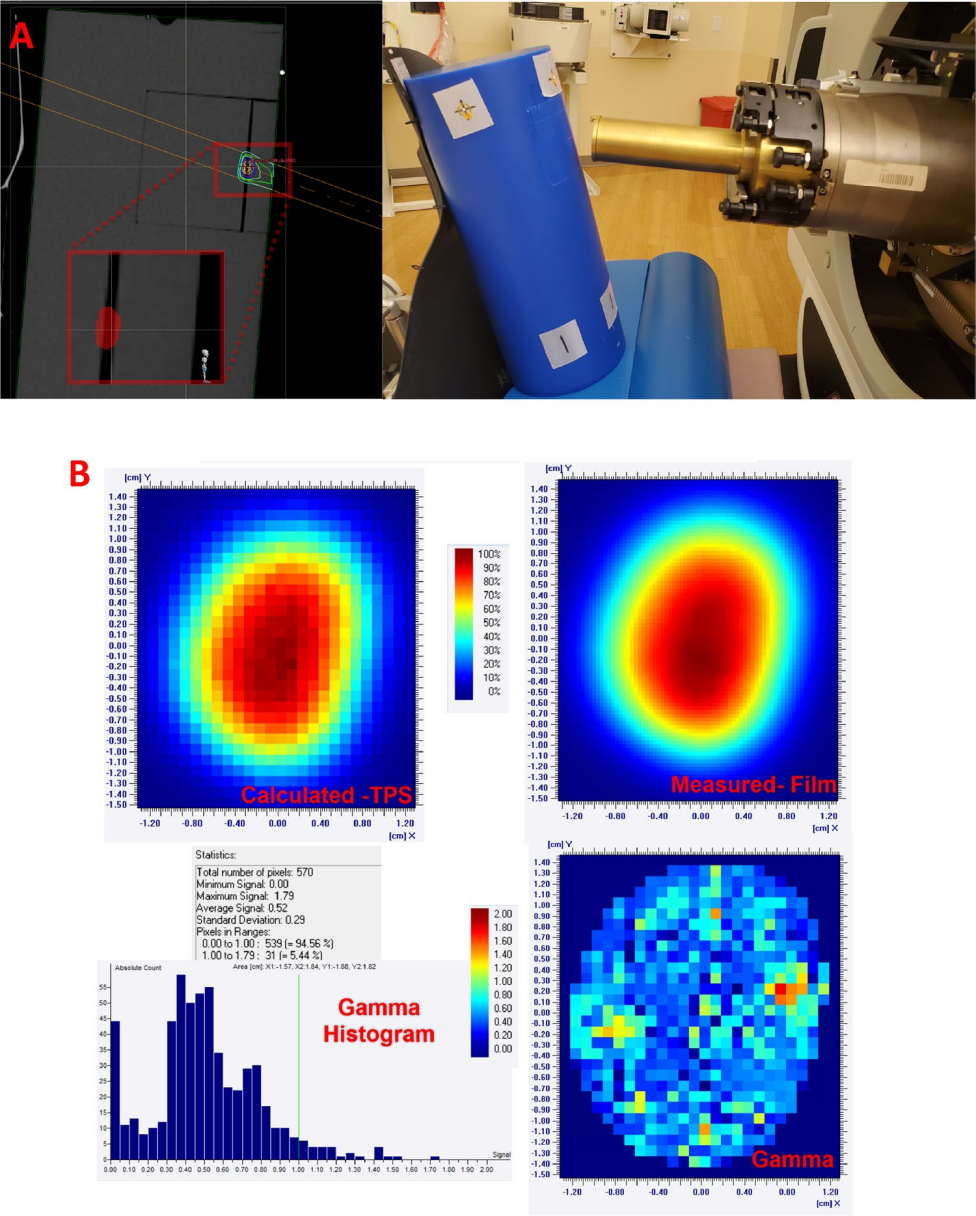
(**A**) End-to-end treatment planning and delivery for a pseudo ocular target (see zoomed inset, target is roughly 0.5 cm width, 0.8 cm length, and 0.4 cm thick at a depth of 2 cm) treated through a single enface beam. (**B**) The comparison of irradiated film with the calculated dose map along with gamma analysis.

The blue wax phantom with embedded EBT3 film was successfully aligned to its treatment position (Fig. 7A) initially using the external BBs and final alignment based on the orthogonal X-rays. The residual alignment errors in the x-, y-, and z-direction were less than 1 mm. Once the treatment was delivered, the resulting film was scanned and converted to dose using the red channel. The comparison of irradiated film with the calculated dose map is shown in Fig. 7B. Using 1% dose difference, 1 mm distance-to-agreement, and 10% low dose threshold, a gamma index of 94.6% was obtained. For point dose measurement, the measured value was 1053.7 cGy against the calculated value of 1050 cGy, within the 3% tolerance.

### Simulated lateral and distal penumbras

**Figure 8:**
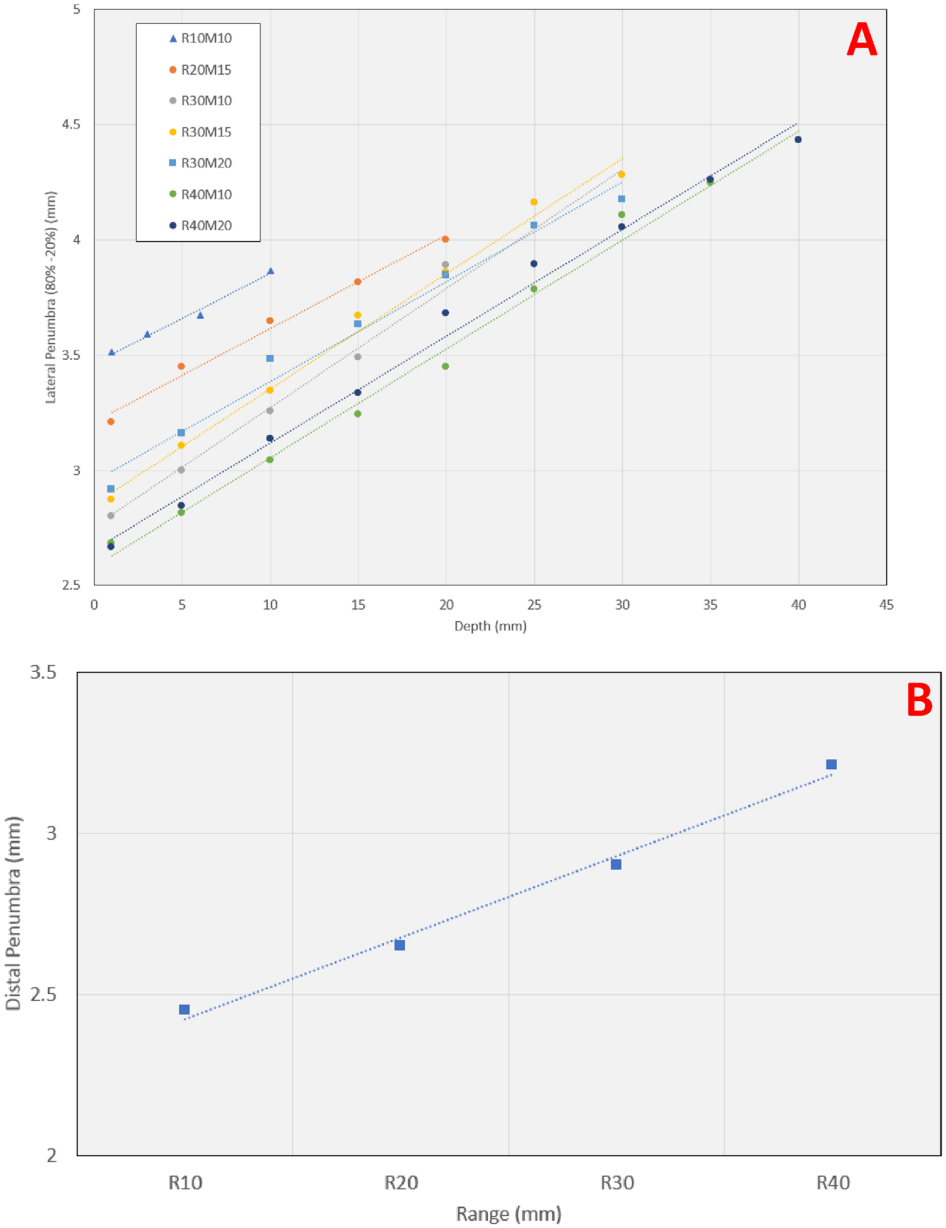
Variation in the (**A**) lateral penumbra with depth for seven beams simulated with various range-modulation combinations (**B**) distal penumbra as a function of the proton beam range. The actual measurement points correspond to marker locations, linear trendlines are shown for visualization purposes.

Figure 8A shows the variation in the lateral penumbra (80–20% dose) with depth for seven beams simulated with various range-modulation combinations. The lateral penumbra ranges from 2.6 to 4.4 mm for all range, modulation, and depth combinations considered. Figure 8B also shows the distal penumbra (80–20% dose) as a function of the proton beam range. There was no variation in distal penumbra with modulation, provided range was kept constant. The distal penumbra varied from 2.45 mm at a range of 10–3.2 mm for a range of 40 mm.

## Discussion

Proton therapy is a proven modality for the treatment of ocular melanoma. Most centers that treat ocular tumors are based on scattering systems with specialized beamlines. However, the new and upcoming centers are based on pencil beam scanning. Most of these centers do not invest in a separate ocular beamline as costs associated may not be offset by the anticipated ocular treatments. There is a need for a system that can convert a conventional beamline into an ocular beamline. Validation and clinical implementation of an ocular applicator that mounts on a conventional PBS snout are presented in this work. Our presented solution allows for PBS-only facilities to treat ocular targets without the need for a specialized beamline or additional commissioning, thus increasing access to ocular proton therapy.

One of the challenges in treating ocular targets are the uncertainties associated with small field dosimetry. We utilized a cross-calibrated microdiamond detector for our measurements. The microdiamond detector has been widely used for proton therapy and its response is shown to be independent of the proton beam energy, direction, and field size^20^. Although the RayStation treatment planning system has been validated extensively for MC dose calculations^7,8,21–23^ most of the validation has been performed for large fields (> 5 cm). The RayStation physics manual also asks users to ensure that field sizes are greater than 4 cm^24^. The ocular field sizes are smaller with the typical field size range at our institution from 1.5 to 2.5 cm. Our work for creating an ocular applicator involved extensive small field dosimetric measurements. All our results for range, point doses, depth doses, and profiles were within our institution’s established clinical tolerance. These results suggest that RayStation’s Monte Carlo dose engine could be used for field sizes smaller than 4 cm for the proton beam ranges considered in this report, without any additional commissioning effort. While this work is focused on ocular treatments, we believe that treatment of field sizes less than 4 cm are also feasible for other disease sites.

Sharp lateral and distal dose fall-offs are key for an ocular beamline to minimize dose to critical OARs. Our ocular applicator yields lateral penumbras in the range of 2.6–3.6 mm (function of energy) at 1 mm depth inside a water phantom. The comparison of penumbra with other published data is difficult as penumbra changes with the simulated beam conditions such as range, modulation, depth, and air-gap between the phantom surface and the edge of the aperture. Quite often penumbra values are given in literature without specifying the relevant beam conditions. Slopesema et al.^11^ provided lateral penumbra values for various proton beams for their dedicated double scattering-based proton beamline. For their range of 10 mm at full modulation and an air gap of 6.5 cm, the reported lateral penumbra is 1.8 mm at the surface of a phantom. The corresponding value of our system is 3.6 mm, implying that the lateral penumbra of our system is larger than their dedicated beamline. Slopesema et al.^11^ also reported the best-case lateral penumbra from 12 different international proton facilities that ranged from 0.9 to 2.2 mm. Our lowest penumbra at the entrance is 2.6 mm, roughly similar to their largest reported value. In another study^18^ conducted at 15 ocular facilities worldwide, the minimum, maximum, and average reported lateral penumbra values were 1.4, 4.3, and 2.6 mm respectively. These results are very similar to our lateral penumbra values. In another study by Ciocca et al.^9^, lateral penumbra values for a non-dedicated beamline in the middle of SOBP ranged from 1.4 to 1.7 mm. Their proton therapy system only required a 28 mm thick WET range shifter allowing for sharper dose fall-offs. They also optimized the position of the range shifter through MC simulations found that range shifter location before the beam monitoring chambers (98 cm upstream of isocenter) to be ideal for minimizing lateral penumbra. However, placing a range shifter upstream of the beam monitoring chamber is not possible for us and many other proton therapy centers as it would require the equipment vendor to approve and validate the beamline. Slopesema et al.^11^ also provided distal dose fall-off for their dedicated eye-lines and other 10 facilities with values ranging from 0.7 to 6.6 mm. The higher values belonged to systems where room energy was higher (≥ 150 MeV) and required significant degradation to create ocular relevant clinical beams. Our distal penumbra values ranged from 2.5 to 3.4 mm in an effective proton beam range of 10–40 mm. We are exploring the design to put the range shifter upstream inside the snout housing to reduce the penumbra further.

Due to its unique design, our applicator allows small air gaps between the patient's surface and the applicator's downstream end without interfering with the patient's field of view. From our clinical experience, the treatments that required 8–10 cm of air gap through conventional snout can be performed with 3–5 cm air gaps with our applicator. Our lateral penumbra roughly increases by an mm for every 2 cm increase in the air gap; thus, the applicator could reduce the lateral penumbra by 2–3 mm for patient treatments.

Based on comparison with published results, both the distal fall-off and lateral penumbra of our beamline are clinically acceptable to yield quality plans. Although our lateral penumbra values are at the higher end of the published results, there are many other advantages to our system. Using inverse planning, our system provides greater flexibility to a planner in shaping the dose away from the critical structures. Pencil beam scanning allows for beamlet level flexibility enabling proximal dose shaping, which is not possible in a scattering-based system. Additionally, the use of MC based dose algorithm means the resulting dose calculations are more accurate as shown by numerous recent publications^7,8,21–23,25^.

## Conclusions

In summary, an ocular applicator for a proton therapy system using pencil beam scanning has been designed and modeled in a commercial treatment planning system. Small field dosimetry for field size less than 4 cm has been validated by comparing depth doses, profiles, and point doses between calculated and measured data for ranges relevant to ocular treatments. Since the discrepancy in the small field dosimetry is within an acceptable tolerance, this validation allows the use of PBS modality to treat ocular tumors. In contrast to forward planning in Uniform Scanning, inverse planning in PBS helps to conform the dose in the proximal edge of the tumor sparing normal tissue proximal to the target. The ocular applicator enables lateral and distal penumbra that is comparable to published values from the other centers.
